# *Artemisia carvifolia* Buch silver nanoparticles downregulate the Rap2A gene in liver cancer

**DOI:** 10.1038/s41598-023-48946-0

**Published:** 2023-12-06

**Authors:** Sabahat Javid, Erum Dilshad

**Affiliations:** Department of Bioinformatics and Biosciences, Faculty of Health and Life Sciences, Capital University of Science and Technology(CUST), Islamabad, 44000 Pakistan

**Keywords:** Cancer, Drug discovery, Plant sciences, Materials science, Nanoscience and technology

## Abstract

Liver cancer is the second main reason of death globally. In the current study, Rap2A protein a member of Ras Gtpase was selected as a drug target for liver cancer which has been identified as an oncogene in different types of tumors. The present study aimed to evaluate *Artemisia carvifolia* Buch extract and its silver nanoparticles against liver cancer targeting the Rap2A gene. The synthesized silver nanoparticles showed an absorbance peak at 450 nm by a UV–Vis spectrophotometer. SEM revealed that polyhedral silver nanoparticles had a size ranging from 80 ± 6 nm. Furthermore, amines, aldehydes, ketones and alcohols of *Artemisia carvifolia* were found involved in the reduction and stabilization of nanoparticles by FTIR. Moreover, XRD and EDX confirmed the cubic crystalline nature and particle elemental composition, respectively. Furthermore, the cytotoxicity against HePG2 cancer cell lines was also found significant with an IC_50_ value of 2.57 µM for silver nanoparticles and 11.57 µM for plant extract. The gene expression and protein level of Rap2A were also decreased in plant extract and nanoparticle-treated cells compared to control groups. The apoptotic potential of extract and nanoparticles was also determined by evaluating the apoptotic pathway genes and protein including BAX, caspase 3, 8 and 9. Significantly elevated levels of expression of these genes by real-time qPCR along with increased protein levels by ELISA were found. This is the first-ever report describing the synthesis and efficacy of silver nanoparticles of *Artemisia carvifolia* Buch against liver cancer.

## Introduction

Cancer is a global threat to human lives that occurs due to the uncontrolled growth of abnormally organized tissues^1^. Recently, based on the reported data, the number of newly diagnosed cancer cases reached more than 19 million, which led to 10 million deaths in 2020^2^. Hepatocellular carcinoma (HCC) is the most common type of primary liver cancer which is the 6th leading cancer in prevalence and the 4th most common source of cancer-related deaths globally^3^. There are many causes of hepatocellular carcinoma, including chronic liver disease, HBV and HCV infection, and nonalcoholic steatohepatitis^4^. Numerous possible biomarkers have been identified by the latest development of high throughput sequencing data for determining the prognosis of patients^5^. Rap proteins belong to the Ras GTPase binding family having 50 to 60% similarity of sequence with RAS protein. The variety and accuracy of both proteins are determined through various parts of GEFs and GAP. In human genes, five special genes of the RAP family such as RAP 1A, 1B, 2A, 2B and 2C have been recognized. RAP proteins mainly play a role in cell adhesion, movement and polarity^6^. The result of RAP gene commencement relies on the context-precise contact of the RAP gene with their monitors and downstream effectors.

The cancerous role of the RAP gene has been recognized in various forms of tumors for instance breast, lung, ovary stomach, cervix, prostate, and brain tumors^7^. Estimated indication advocates that RAP proteins also take part in serious functions in HCC and cancer development. Single nucleotide polymorphism in the RAP1A gene (rs494453) has been represented to be linked among high occurrence and reappearance after transplantation of the liver. More advanced action of the NF-κB/RAP1 signaling channel is linked to tumorigenicity in hepatocellular carcinoma^8^. There has been a strong link between liver inflammation and RAP1A expression, which is a risk factor for liver carcinogenesis^9^.

Nanotechnology is one of the newest scientific achievements leading to rising industries with a public and financial interest. This felid is a modern form of material manufacturing by handling and operating material structures on a nanoscale^10^. The major purpose of most nanoscience studies is to manufacture novel resources or to formulate accessible materials. Nanoparticles show better or considerably enhanced properties based on definite characteristics for example morphology, size and distribution. Bulk and atomic structures vary in their characteristics and metal nanoparticles bridge the gap among them with their exclusive physicochemical properties, which are more surface area, more reactivity, large surface-to-volume ratio, spatial confinement, tunable pore size and particle morphology. The nanoparticles made up of inert metals mainly Au, Ag, Pt and Pd are investigated efficiently in medical applications against various diseases including cancer^11^.

At the commercial levels, silver metal is extensively used in nanotechnology which contributes 5 hundred tons for synthesis of silver nanoparticles annually^12^. Silver nanoparticles (AgNPs) have prominent role in wound healing due to their innate therapeutic properties^13^. They also play a prominent role in therapeutic applications, such as through anticancer, antidiabetic, antioxidant, antimicrobial, and antiviral activities^14^. Green synthesis of nanoparticles using ecofriendly substances is a developing branch in nanotechnology. Recently, inspired by the perception of green chemistry, the biological synthesis of nanoparticles has been focused on utilizing biological entities, such as plants, algae, and micro-organisms. It offers many advantages over chemical synthesis, such as environment-friendly, cost effectiveness, efficient energy utilization, and suitability for biomedical and pharmaceutical applications. Plants are considered to be a superior source for the synthesis of nanoparticles as compared to chemical synthesis, as they are eco-friendly and nontoxic^15,16^.

The genus *Artemisia L.* belonging to the *Asteraceae* family exhibit efficient and therapeutic implication. Plants of this group are frequently found in the moderate sectors of the northern hemisphere with an inadequate number of species in the southern hemisphere of the globe^17^. It contains approximately 500 species of both herbs and shrubs and is a different genus of the Anthemideae tribe^17,18^. The financial significance of numerous plants of *Artemisia* species is because of their consumption as aesthetics, feedstuff, fodder, therapeutics, and soil binder, although some species are allergic and toxic weeds^17^. The artemisia genus is reported to have medicinal properties including antioxidant, anti-inflammatory, antimicrobial and with numerous anti-cancer compounds present in it^19^. *Artemisia carvifolia* Buch has been reported to have antidiabetic, antimalarial and anticancer properties. It has also been reported to have phytoconstituents including flavonoids, artemisinin and derivatives with anticancer properties^20–22^.

In the proposed study, we observed the effect of *Artemisia carvifolia* Buch extract and its silver nanoparticles on the Rap2A gene for countering liver cancer progression. The study involved dose-dependent treatment of human liver cancer cell lines with plant extract and silver nanoparticles. Expression of Rap2A and downstream targets of the apoptotic intrinsic pathway were determined both at gene and protein levels. These attempts were meant to unlock the mechanisms behind the natural control of human liver cancer and will continue to be an important insight in the continuous search for an effective cancer drug design and management. This is the first-ever report of green synthesis of silver nanoparticles using *Artemisia carvifolia* Buch.

## Experimental

### Collection and identification of plant material

*Artemisia carvifolia* Buch seeds obtained commercially were germinated^23^ and DNA barcoding was done for plant identification using *psbA-trnH* region of chloroplast genome^24^. Plant specimen were submitted in the herbarium of Quaid-i-Azam University Islamabad, Pakistan with specimen voucher no. HMP-ART 0001. All the experiment was done in accordance with the institutional, national and international guidelines for conducting plant research.

### Silver nanoparticles preparation and characterization

*Artemisia carvifolia* Buch plant extract of different concentrations was prepared (10, 20, 40, 80, 160 mg/mL)^25^. Similarly, a 5 mM solution of AgNO_3_ was made. Afterwards, both were mixed in a ratio of 1:9 and put under sunlight for 12 h which lead to changing the solution colour to blackish brown. Characterization of synthesized silver nanoparticles by UV–Vis, SEM, EDX, XRD and FTIR was done according to the reported method^26,27^. UV–Vis analysis was done by using UV–Visible spectrophotometer (UV 1602 BMS spectrophotometer, Spain). This was done by placing reaction mixture in a quartz cuvette, and the absorbance was measured from 300 to 600 nm^28^. Furthermore, size and morphology details were attained by SEM analysis (JEOL-JSM-6490LATM) operating at the voltage of 20 kV (maximum) with the counting frequency of 2368 cps (maximum). Chemical composition was confirmed by EDX (Oxford instruments) coupled with the SEM as plugin hardware. For that purpose, dried AgNPs were mounted on carbon tape and coated with gold sputtering for 2 min and then analyzed^28^. The surface chemistry of synthesized nanoparticles was studied by the FTIR spectrometer. The AgNPs solution was dried at the temperature of 75 °C and the dried powder of AgNPs was subjected to characterization in the range of 4000–400 cm^−1^ utilizing a KBr pellet strategy. The samples for X-ray diffraction (XRD) analysis were made by taking a small amount of solution from the bottle and drying it on a quartz plate (XRD D8 Advance, Bruker, Germany)^26,28^.

### MTT assay

The cytotoxic potential of synthesized nanoparticles was tested by MTT assay against liver cancer HePG2 cell line (obtained from ATCC with ATCC number HB-8065™) according to the reported procedure^21,22,29^. Briefly, DMEM was used for cell culturing which was added to 96 well flat bottom plates in triplicate. The wells were added with the different concentrations (50, 40, 30, 20, and 10 µM) of prepared nanoparticles and plant extract. Afterwards, incubation was done for 24 h at 37 °C with 5% CO_2_. This was followed by the addition of 10 µL MTT (5 mg/mL) incubated at 37 °C for 3 h. Finally, solubilization solution (100 µL) was added to the wells and incubated for 2–4 h in the dark. Lastly, the absorbance of the samples was measured at 570 nm in plate reader FLUO star Omega (BMG Labtech). IC_50_ was calculated.

### Determination of expression of target genes by real-time qPCR

Determination of expression of target gene Rap2A and apoptosis regulatory protein Bax along with caspases (caspase 3, caspase 8 and caspase 9) were determined by real-time qPCR by following the reported methodology^9,30^. Briefly, RNA was isolated from all the treated and control cells and complementary DNA (cDNA) was synthesized. The gene-specific sets of primers for Rap2A, Bax, caspase 3, caspase 8, caspase 9 and housekeeping gene GAPDH^30^ were used as reported earlier^9,30^.

### Determination of the level of proteins by ELISA

ELISA kits were used to study the level of proteins of target genes Rap2A, Bax and caspases enzymes based on the manufacturer’s protocol for Rap2A (Abbexa), BAX (Invitrogen) and caspases (Thermo-Fisher). Shortly, the lysates of cells were made ready according to the instructions of the ELISA kit. Cell lysate proteins explicitly bound to the primary antibody and were spotted by a secondary antibody conjugated with Horseradish peroxidase. Afterwards, a microplate reader was used to measure protein levels at the absorbance of 450 nm.

### Statistical analysis

All the data was obtained in triplicate, and the values are presented as the mean ± standard error of the mean. Two-way ANOVA was used to analyze the collected data statistically using the GraphPad Prism (V.5) software. The *p* value of less than 0.05 was considered statistically significant.

## Results and discussions

### Plant identification

After amplification of *psbA-trnH* region (500 bp) of chloroplast genome and sequencing of amplified region of DNA, BLAST in NCBI and CLUSTAL W in BioEdit software (version 7.2.5.0) was performed. Reference sequence (GenBank Accession number [NCBI: FJ418751]) was used for the identification of plant under study. It was confirmed it to be *psbA-trnH* sequence of *Artemisia carvifolia* Buch^24^.

### Synthesis and characterization of silver nanoparticles

**Figure 1:**
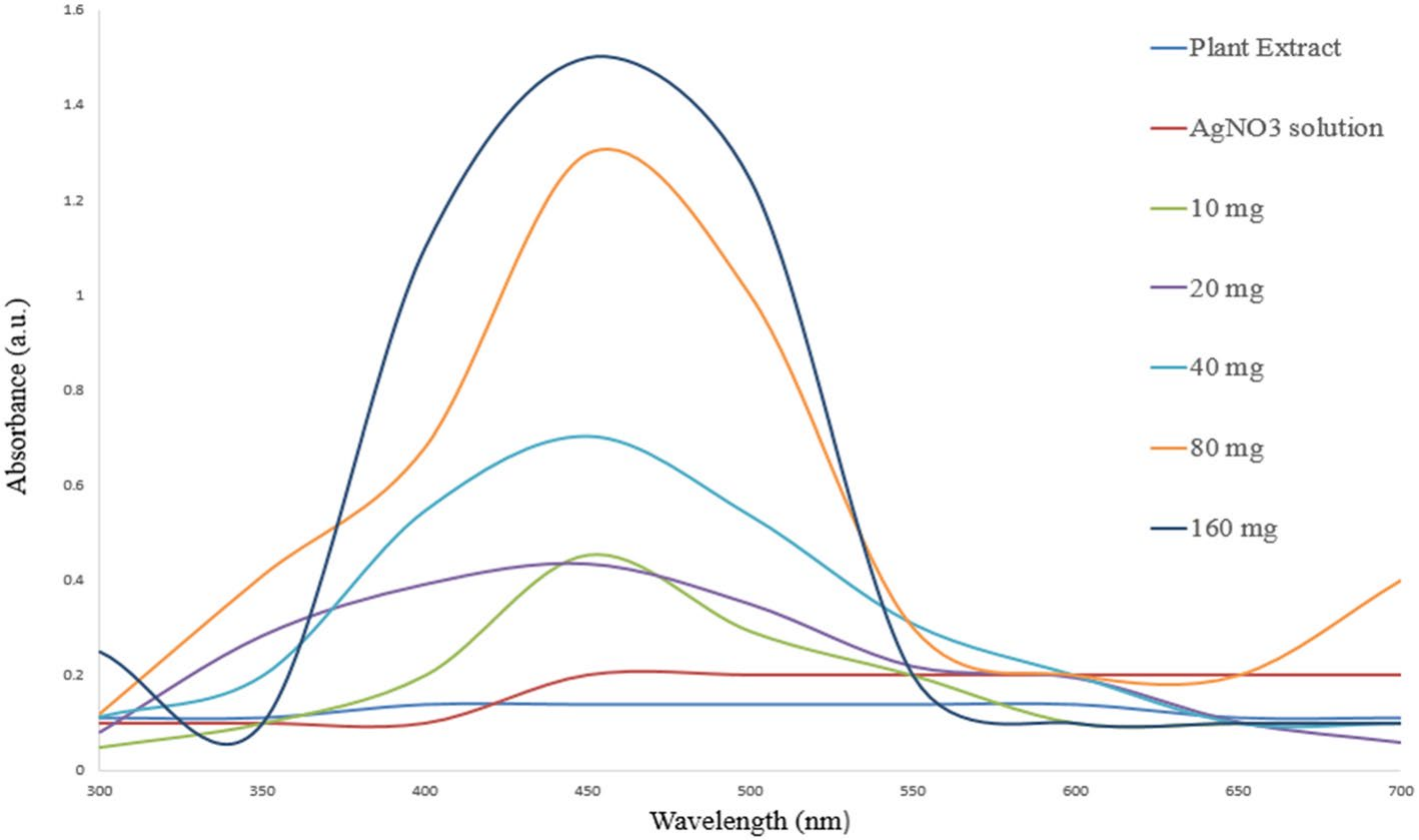
UV–Vis spectroscopy of synthesized silver nanoparticles. The spectra show the absorption peaks of nanoparticles synthesized by different concentrations of plant extract at 450 nm.

Silver nanoparticles synthesis was done successfully by mixing plant extract and silver nitrate salt solution. The colour change from yellowish to dark brown indicated the complete formation of silver nanoparticles^31^. Further confirmation was done using UV–Vis spectroscopy with a strong peak at 450 nm^27^. SPR (surface plasmon resonance) is responsible for UV– Visible maximum absorption in the range of 400–500 nm^32^. Different concentrations of *Artemisia carvifolia* extract 10, 20, 40, 80 and 160 mg/L were used to optimize the synthesis of silver nanoparticles. There was observed 1.5 times increase in the absorbance intensity with an increase in the concentration of the plant extract with the highest absorbance at 160 mg/L (Fig. 1).

**Figure 2:**
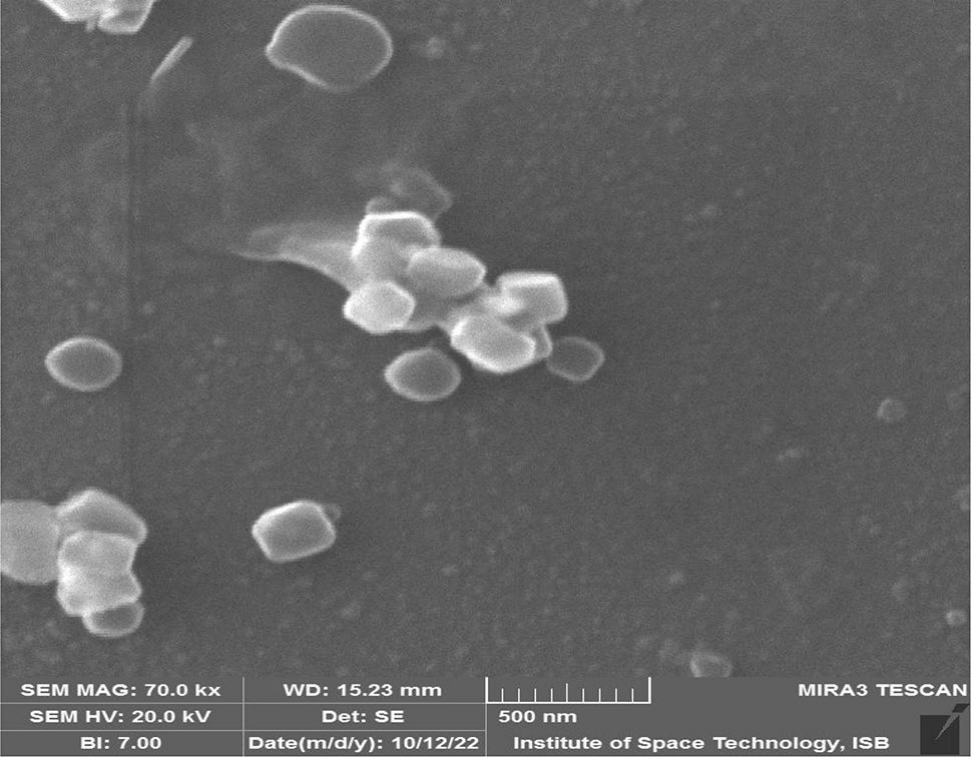
SEM analysis of silver nanoparticles: The images are presenting the morphology of the synthesized nanoparticles. Scale bar = 500 nm.

These findings are also supported by other reports^33^. The proportion of silver ions to their capping and stabilizing agents determines the extent of silver nanoparticles. Moreover, the reducing agent’s concentration and that of the metal itself also hold a vital part in the synthesis of silver nanoparticles. In an earlier report, comparable results were also described in the silver nanoparticles of *Artemisia annua*^34^. Scanning electron microscopy (SEM) gave additional understanding into the size and morphology of the green synthesized silver nanoparticles. The nanoparticles were found with a calibrated size of 80 ± 6 nm. Furthermore, the shape of silver nanoparticles was found to be icosahedron (polyhedral) (Fig. 2).

**Figure 3:**
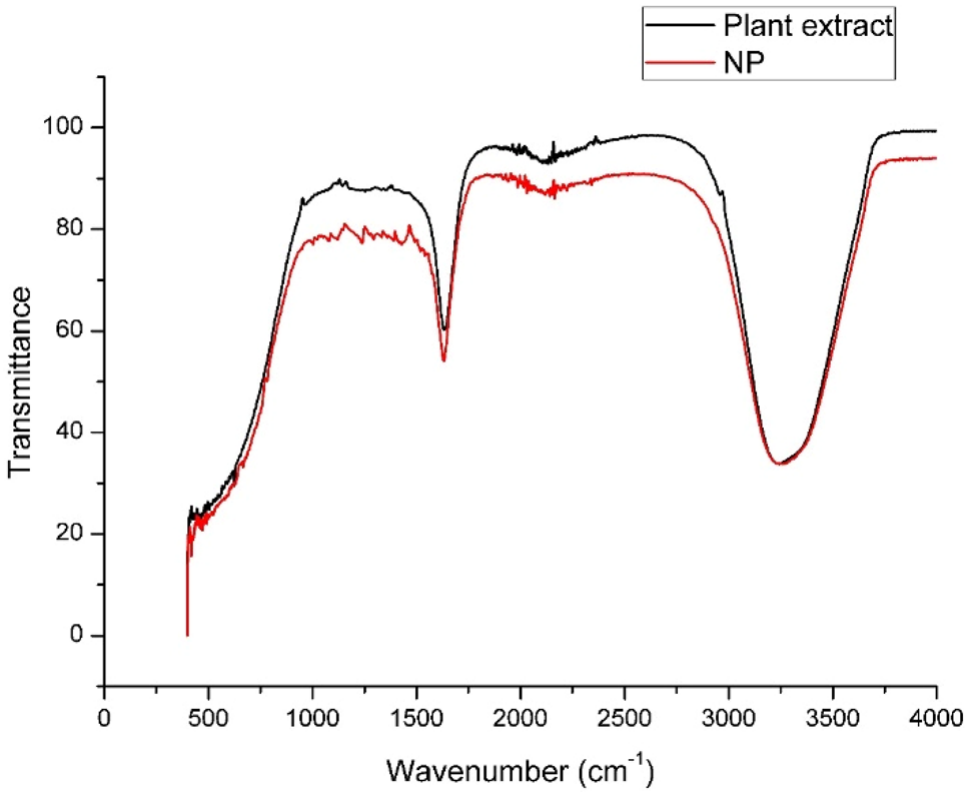
FTIR of synthesized silver nanoparticles and plant extract.

FTIR was carried out to find the latent natural compounds in *Artemisia carvifolia* Buch plant extract which were involved in the synthesis of nanoparticles. The spectra depicted changes between 800 and 1500 cm^−1^ which confirmed the involvement of flavonoids, phenolics and lipid-containing oils having aldehydes and ketonic bonds (Fig. 3). Ketones with carbonyl (C–O) stretching vibration were observed at 1000–1140 cm^−1^. It was confirmed from FTIR investigations, that the amino acid and proteins resulting in carbonyl gatherings have the extra beached capacity to bind with metal, displaying that the proteins might be involved in shaping the metal nanoparticles. Previously, silver nanoparticles of *Artemisia marschalliana* showed similar peaks showing the vibrations of carbonyl (C–O)^35^. Similarly, the = C–O stretching between the 1100–1350 cm^−1^ may go with the lipid’s carboxyl groups. Similar findings were observed for nanoparticles of *Artemisia annua*^34^. There was observed some change in the range of 1331–1334 cm^−1^ showing the involvement of carboxyl groups in the synthesis of the silver nanoparticle.

**Figure 4:**
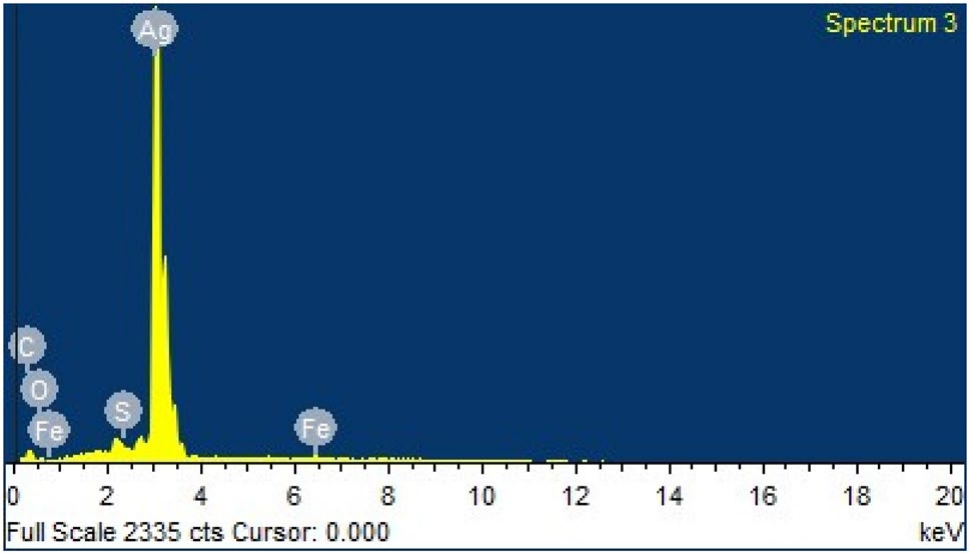
Energy-dispersive X-ray spectroscopy (EDS) of synthesized silver nanoparticles showing silver (Ag) as the major component of nanoparticles.

**Figure 5:**
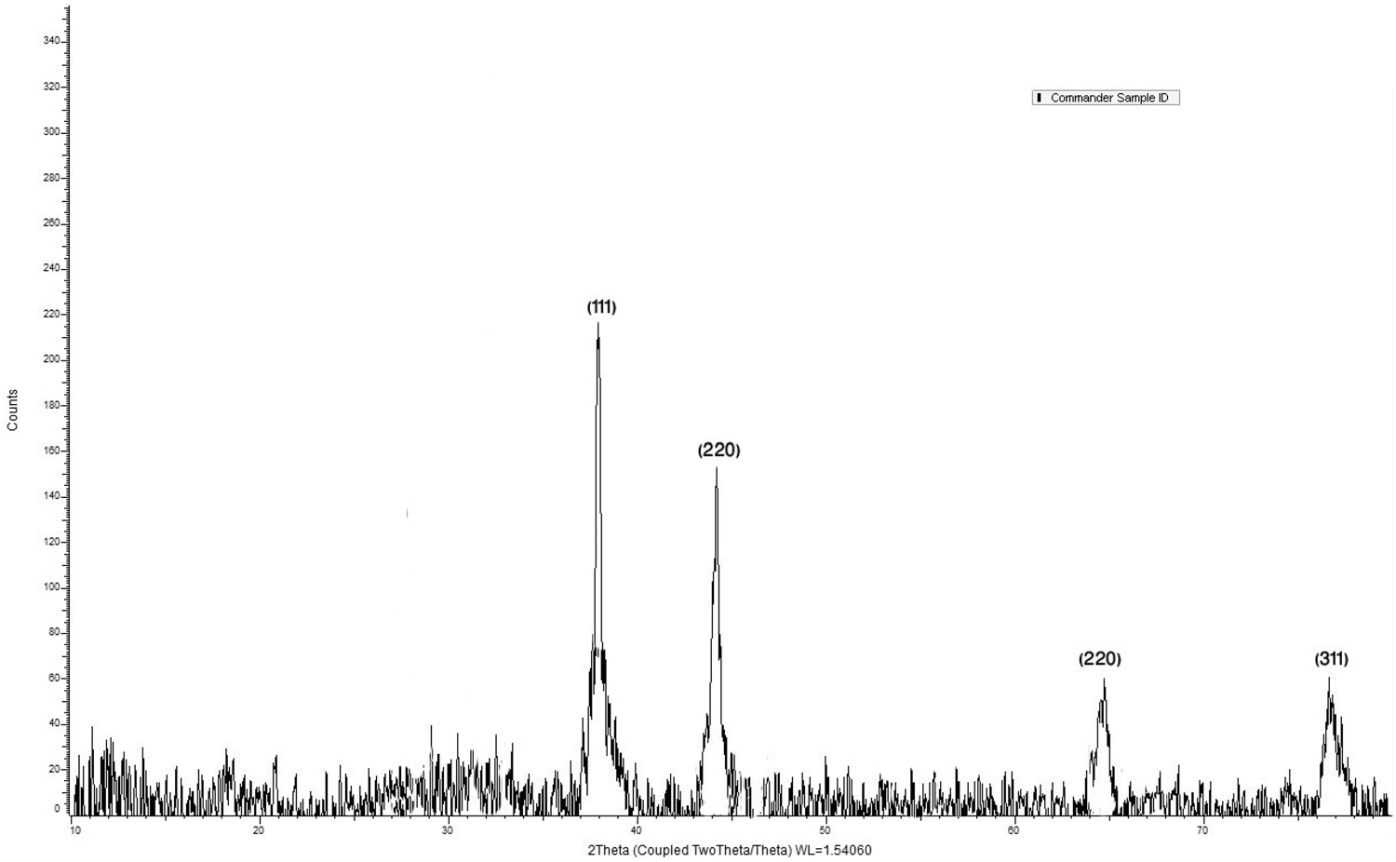
X-ray diffraction (XRD) analysis of synthesized silver nanoparticles. The spectrum shows face-centered cubic (FCC) crystalline metallic silver nanoparticles. The intensity on the vertical axis is measured in counts per second (CPS), and the diffraction angle (2 theta) measured is taken along the horizontal axis. The value of wavelength in angstrom (a.u.) is also indicated.

Moreover, EDX was used to study the elemental composition of silver nanoparticles (Table 1) which showed silver as the major element (Fig. 4)^26^. X-ray crystallography results showed the crystalline nature of silver nanoparticles with identified peaks [38.23 (1 1 1), 44.41 (2 0 0), 64.38 (2 2 0), 77.5 (3 1 1)] at 2θ° (Fig. 5). These findings were found in agreement with the standard ICSD No. 98–018-0878^14,27^.

**Table 1:** Elemental composition of EDS spectrum.

Element	Weight%	Atomic%
C K	2.92	16.64
O K	5.84	24.97
S K	0.15	0.31
Fe K	0.59	0.72
Ag L	90.50	57.36
Totals	100.00

### Cytotoxicity assay

**Figure 6:**
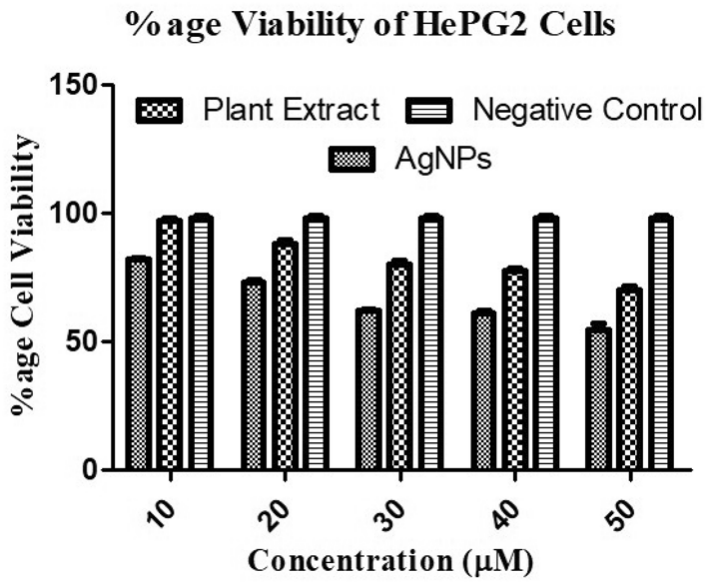
Antiproliferative activity of silver nanoparticles: Percentage (%) cell viability of HePG2 cell line after treatment with silver particles and plant extract.

To study the cytotoxic potential of silver nanoparticles, samples were tested against liver cancer cell lines HepG2 (Fig. 6). In this study, four different concentrations of synthesized silver nanoparticles and plant extract (10, 20, 30, 40 and 50 µM) were used to study their cytotoxicity. Percentage cell viability of HePG2 cells was found to be decreased more at the higher concentration (50 µM) than at lower concentration (10 µM). Plant extract and silver nanoparticles showed cytotoxicity in a concentration dependent manner indicating their increased cytotoxicity at higher concentration. Furthermore, IC_50_ of silver nanoparticles was found 2.57 µM for HepG2 cells, while for plant extract it was 11.57 µM. The statistical significance of the data was also observed (Table 2).

**Table 2:** Analysis of variance for factors affecting the viability of HePG2 Cells.

Source of variation	Df	Sum-of-squares	Mean square	F-Value	*p* Value	Significant
Interaction	8	1122	140.3	12.11	< 0.0001	Yes
Types of nanoparticles	2	10,330	5163	445.9	< 0.0001	Yes
Concentration	4	561.0	140.3	12.11	< 0.0001	Yes
Residual	30	347.3	11.58			

Previously synthesized silver nanoparticles of *Artemisia marschalliana* were found active when tested against the cells of gastric carcinoma^35^. Another report of synthesized biogenic silver nanoparticles of the copperpod plant showed significant cytotoxicity against MCF7 and HePG2 cell lines^36^. Previously, green synthesized silver nanoparticles have been shown to exhibit anticancer activity against MCF7 and HePG2 cell lines, while, silver oxide nanoparticles were found cytotoxic to HePG2 and Chang liver cells^37^. In another study, we prepared maleic acid- and citric acid-capped silver particles and they were reported to have an inhibitory role against liver cancer (HePG2)^27^ and those synthesized by green chemistry approach using extract of *Mentha asiatica* were also found to have similar results^26^.

**Figure 7:**
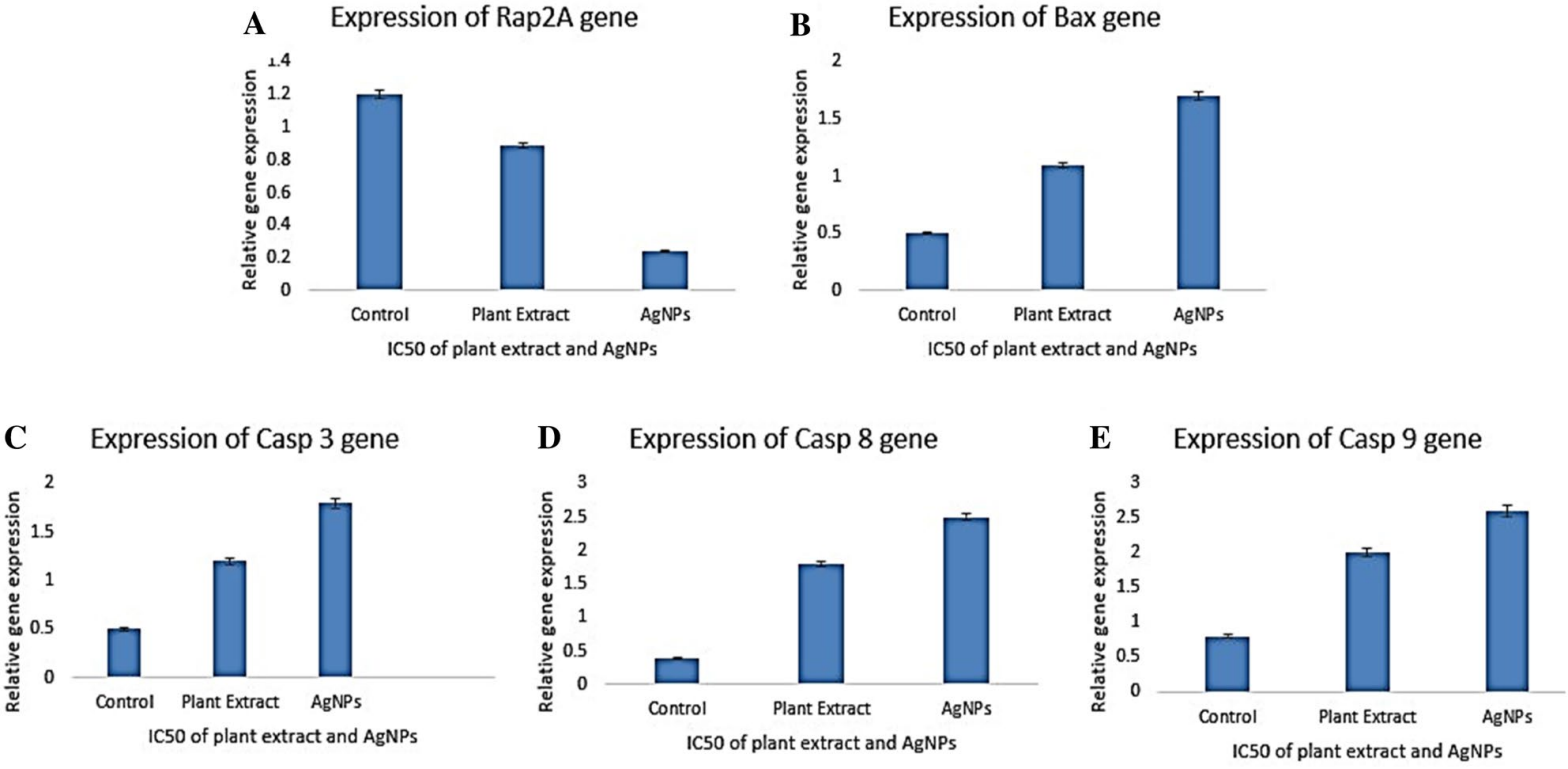
Gene expression studies by Realtime-qPCR, showing the level of expression of Rap2a (**A**), Bax gene (**B**), caspase 3 (**C**), caspase 8 (**D**), and caspase 9 (**E**).

The level of expression of the target gene of liver cancer (Rap2A) in silver nanoparticles and plant extract-treated HePG2 cells was found to be significantly decreased as compared to untreated cells indicating its role as an oncogene in liver cancer (Fig. 7A). The experiment was run with the IC_50_ of plant extract and synthesized silver nanoparticles and it was observed that expression of Rap2A gene was decreased more prominently in HePG2 cells treated with synthesized silver nanoparticles than that of plant extract. Previously, the expression of RAP genes in HCC was studied by detailed molecular and clinical features. In that study, there was observed a strong association of Rap2A gene in HCC by pathway analysis including both metabolic pathways and those related to cell cycle. Where it was observed that RAP2A showed a robust capability to differentiate tumors from normal tissues. The expression of RaP2A was related to the modification of its copy numbers and DNA methylation^7^.

Furthermore, the stimulatory effects of synthesized silver nanoparticles and plant extract on the Bax gene up-regulation were also studied (Fig. 7B). Similarly, experiment was run with the IC_50_ of plant extract and synthesized silver nanoparticles. Synthesized silver nanoparticles caused more upregulation of Bax gene in HePG2 cells than that of plant extract. High levels of expression of the Bax gene suggested the role of silver nanoparticles in HePG2 cell apoptosis through an intrinsic apoptotic pathway. In a previous study, similar findings were observed, where silver nanoparticles significantly increased Bax gene expression in the liver cancer cell line. It was also reported that the relative level of the dimerization pattern of the Bax protein shifts the cell to survival or cell death^30^. Moreover, caspase-3, caspase-8 and caspase-9 genes were also evaluated for relative expression to determine the apoptotic role of synthesized silver nanoparticles against treated cells. It was found that silver nanoparticles brought up-regulation of all studied caspase genes expression in HepG2 cells (Fig. 7C-7E).

Apoptosis is a process of programmed cell death and there are different genes involved in the regulation of this process including upregulation or activation of Bax gene expression and death caspases. BAX (key gene in extrinsic IL-3 mediated apoptosis cascade), CASP 3 (the chief executioner of programmed cell death in extrinsic and intrinsic signalling cascade), CASP 8 (starter gene in TNF-α apoptosis cascade) and CASP 9 (starter gene in intrinsic apoptosis cascade) genes showed increased expression in HepG2 cells as compared to control group signifying the cytotoxic role of synthesized nanoparticles in the activation apoptosis pathway.

**Figure 8:**
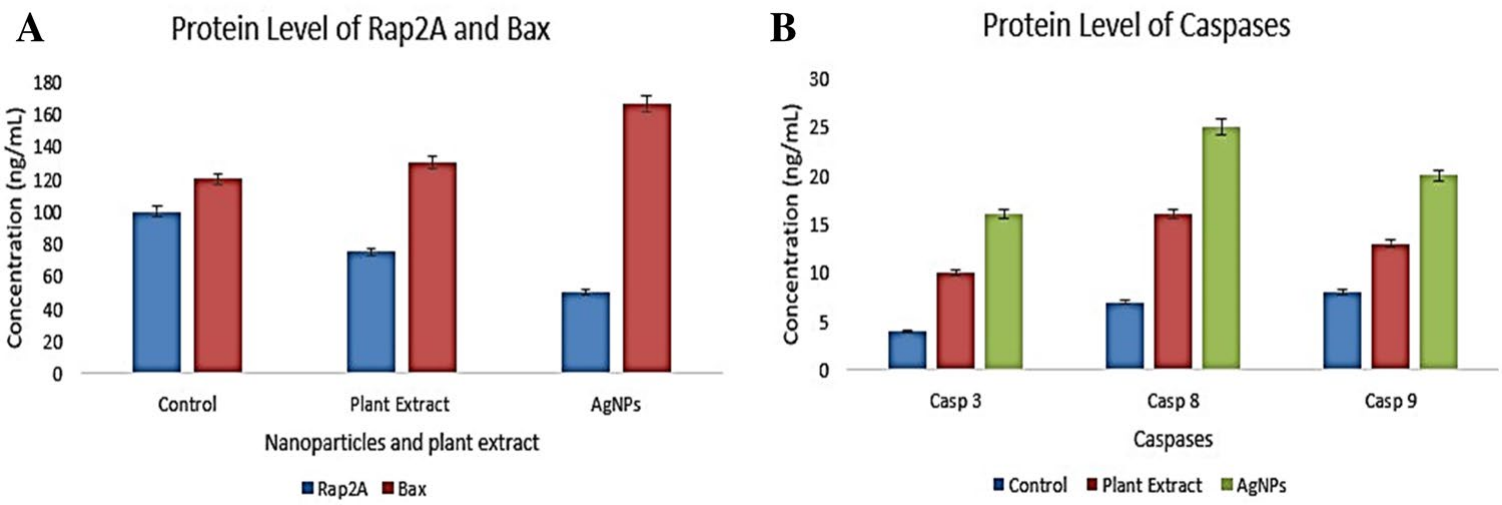
Protein levels determination by ELISA: showing level of Rap2A and Bax proteins (**A**), and Caspases (**B**).

Furthermore, the level of proteins of all studied genes was also evaluated and similar findings were observed which confirmed the activation of programmed cell death of nanoparticles treated HepG2 cells^30^. The Rap2A protein was found to be decreased in plant extract and silver nanoparticles treated cells, whereas the level of Bax and caspase-3, caspase-8 and caspase-9 proteins was found to be increased in the HePG2 cells treated with silver nanoparticles and plant extract. Likewise, synthesized silver nanoparticles caused more downregulation of Rap2A protein and more upregulation of apoptotic proteins than plant extract (Fig. 8A & B). Caspases are regulators of apoptosis and inflammation, having a critical role in the pathways of cell death. In this study, the synthesized silver nanoparticle’s effects were evaluated in the cascades of caspases, as they are the main executioners of apoptosis and cell death pathways in signal transduction^38^. Certain precursors are cleaved by the effector caspases -3/-7 resulting in cell apoptosis. These effector caspases are activated by caspases -8 and -9^39^.

## Conclusion

The *Artemisia carvifolia* Buch plant extract and respective silver nanoparticles were found effective drug candidates for liver cancer targeting the Rap2A gene. Amazingly, silver nanoparticles displayed significant cytotoxic potential against liver cancer cell lines. Rap2A gene expression was found to be downregulated in liver cancer cell line after treatment with silver nanoparticles and plant extract. Furthermore, apoptotic role of synthesized silver nanoparticles and plant extract was confirmed by higher gene expression of apoptotic pathway genes and proteins. The efficacy of synthesized silver nanoparticles was found more than the plant extract. Thus, the current study also provides evidences for using Rap2A gene as potential drug target for treatment of liver cancer. However, further studies are needed to be done in this regard for confirmation in animal models.

## Data Availability

All data generated or analyzed during this study are included in this published article.
